# The Relationship Between Serum Uric Acid at Different Concentrations of Lipid Indices and the Risk of Myocardial Revascularization in Patients With Acute Coronary Syndrome: A Retrospective Analysis

**DOI:** 10.3389/fcvm.2021.732715

**Published:** 2021-08-23

**Authors:** Yajuan Lin, Tesfaldet Habtemariam Hidru, Rui Fan, Jinghan Gao, Han Li, Xiaolei Yang, Yunlong Xia

**Affiliations:** Department of Cardiology, First Affiliated Hospital of Dalian Medical University, Dalian, China

**Keywords:** serum uric acid, myocardial revascularization, lipid indices, ACS, risk factors

## Abstract

**Objective:** Both serum uric acid (SUA) levels and lipid components, such as LDL, HDL, and Lp(a), have been reported to associate with CAD. However, the influence of SUA status at different concentrations of lipid indices for the risk of myocardial revascularization (MRT) in ACS patients is currently unknown.

**Methods:** We retrospectively analyzed a hospital-based sample of 14,234 ACS patients with no previous history of percutaneous coronary intervention (PCI) or coronary artery bypass graft (CABG) surgery. All patients went for coronary angiography. Binary logistic regression models were performed, and the odds ratios (OR) at 95% confidence interval (CIs) were used to approximate the associated risk of UA and lipid profile for myocardial revascularization, with the lowest quartile/tertile serving as the reference category.

**Results:** Overall, 8,818 (61.9%) patients undergone MRT out of 14,234 patients. Elevated SUA and HDL were negatively associated with an increased likelihood of MRT during admission (*P* < 0.001). However, LDL and Lp(a) levels were positively associated with MRT among ACS patients. Furthermore, interaction analyses between SUA and lipid profiles, particularly LDL and Lp(a), compared with those in the lowest quartile of SUA levels, show that patients in higher SUA quartiles grouped by lipid components had a significantly lower chance of undergoing MRT, with the lowest OR (95%CI) for subjects being 0.222 (0.170-0.290), 0.478 (0.374-0.612), and 0.604 (0.468-0.780) in LDL tertiles, being 0.671(0.523-0.862), 0.316(0.242-0.413), and 0.410 (0.310-0.542) in Lp(a) tertiles, respectively. In the three tertiles of HDL levels, the incidence of MRT dropped steadily as SUA levels increased. Also, we further analyzed ACS patients without diabetes. Compared with the first quartile of SUA levels, the risks of MRT were significantly lower in different tertiles of lipids components [LDL, Lp(a), HDL].

**Conclusion:** An increase in SUA levels may decrease the chance of undergoing MRT in ACS patients, even in those with increased Lp(a) and LDL-c. Elevated serum uric acid may play a protective role during an acute stage of ACS.

## Introduction

Coronary artery disease (CAD) is one of the main causes of death all over the world, especially in Asian countries, where its morbidity and mortality are higher than those in Western countries (1, 2). Though recent advances in drug therapy and revascularization have significantly reduced the mortality associated with coronary artery disease, still more is required from the scientific community. Up-to-date, the involvement of a variety of risk factors in the pathogenesis and development of CAD is recognized. Of the already identified risk factors, the status of uric acid (UA) and lipid indices are gaining momentum.

Serum uric acid (SUA) is an end product in the degradation of the purine nucleotides adenine and guanine, and its levels in plasma have been shown to be increased in CAD (3–6) and associated with hypercholesterolemia (7–10). In previous studies, it was suggested that elevated SUA is associated with demonstrable antioxidant activity (11–13). As a potent scavenger of free radicals, the concentration of SUA rises in the condition of acute and chronic oxidative stress. However, many studies revealed the potentially harmful effects of UA (e.g., local tissue damage), partly mediated by an increase in free radical activity (14, 15). Consequently, UA is more known for its role in the development of CAD (3, 16, 17), and its CAD-related adverse outcomes (5). However, a recent study revealed that UA restores endothelial function in patients with type 1 diabetes and regular smokers (18). Also, another study observed an increase in serum antioxidant capacity in healthy volunteers following a systemic UA administration (19). Importantly, in acute myocardial infarction, acute oxidative stress has been observed, in which the subsequent rise of SUA concentration reaches maximal concentrations within a short interval of time (several minutes or hours) (20). This indicates that there is ongoing controversy as to whether the acute increase in UA itself is sufficient to implicate a potential protective benefit during acute coronary syndrome (ACS) or the effect of such an acute increase of UA can be affected by the lipid status of the patients during ACS.

According to a Mendelian randomization study, lipid concentrations including lipoprotein (a) [Lp(a)], low-density lipoprotein (LDL), and triglyceride-rich lipoprotein/residual lipoprotein cholesterol were speculated to participate in the pathogenesis of coronary heart disease (21, 22). Considering the close relationship between UA and other conventional cardiovascular risk factors (such as hyperlipidemia, obesity, hypertension, and diabetes) and the strong antioxidant capacity of SUA, the relationship between SUA and acute coronary stenosis (ACS) is complicated (16, 23, 24). Besides, to the extent of our knowledge, there is no data that report the interaction of SUA and the different levels of lipid indices in myocardial revascularization (MRT). Therefore, this study aimed to explore the relationship between UA/lipid panel and MRT, and whether SUA predicts the risk of myocardial revascularization at different lipid levels in ACS patients.

## Method

### Population Study Design

This population-based retrospective cohort study was conducted between January 2011 and July 2020 at the cardiovascular department of the First Affiliated Hospital of Dalian Medical University (FAHDM), Dalian City, Liaoning Province of China. The clinical data for this study were retrieved from the Electronic Medical Record Research Database (EMRRD) of FAHDM. The EMRRD is established to create a computerized clinical archive, where clinical notes are regularly updated.

**Figure 1:**
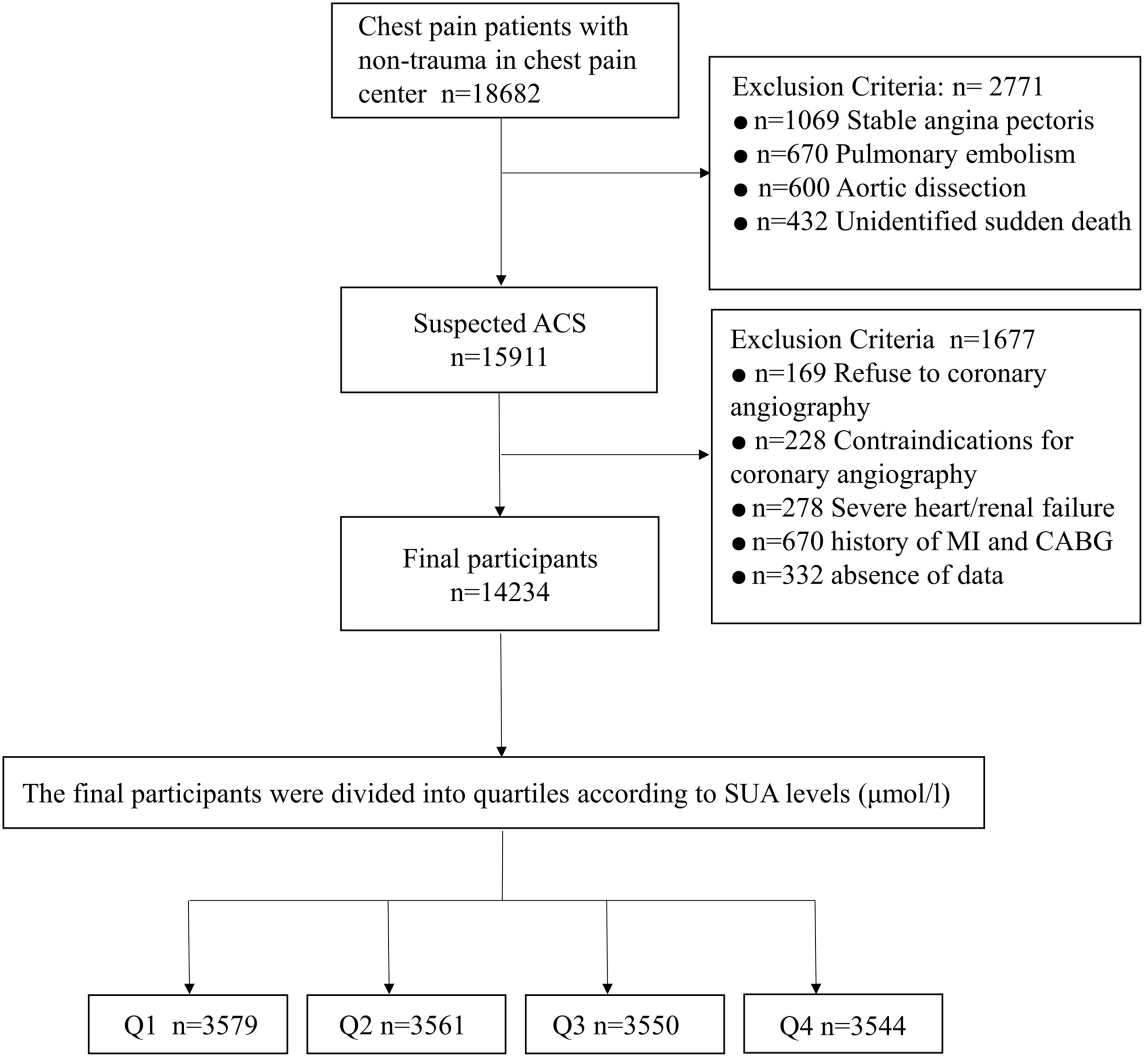
Flow chart.

The present study recruited patients with chest pain who satisfy a minimum age of 18 years with no previous history of percutaneous coronary intervention (PCI) or coronary artery bypass graft (CABG) surgery, and had a complete record of physical examination with complete coronary angiography data. In the first phase of the selection of the participants, we consecutively recruited 18,682 adults who presented with chest pain related to the non-trauma cause. In the second phase, we excluded patients with stable angina (*n* = 1,069), pulmonary embolism (*n* = 670), aortic dissection (*n* = 600), and unidentified sudden death (*n* = 432), and screened a total of 15,911 patients who were eligible for ACS diagnosis. Further, we excluded patients who refused to perform coronary angiography (*n* = 169), had a severe heart/renal disease (*n* = 278), had incomplete data (*n* = 332) on key clinical covariates, had a history of MI (myocardial infarction)/CABG (coronary artery bypass graft) (*n* = 670), and were contraindicated for coronary angiography (*n* = 228). Finally, this study included a total of 14,234 ACS patients. Figure 1 depicts a moderate summary of the study participants' selection. The research was carried out in conformity with the standards of the Helsinki Declaration. The requirement for informed consent was waived as the data was generated from the electronic database of the hospital and the study was approved by the FAHDMU institutional review board.

### Definition of Explanatory Variables

Electronic health records were used to obtain information on demographic and clinical factors such as age, gender, lifestyle, and medication use. The blood samples were collected before coronary angiography at admission and were biochemically tested for blood glucose level, blood cell counts, and serum concentrations of total cholesterol (TC), triglycerides, high-density lipoprotein cholesterol (HDL-c), lipoprotein(a) [Lp(a)], and low-density lipoprotein cholesterol (LDL-c), SUA, and creatinine at the FAHDMU laboratory using the standard protocols. Hypertension was defined as systolic blood pressure at least 140 mmHg, or DBP at least 90 mmHg, and/or a self-reported history of hypertension with/without antihypertensive medication. Diabetes mellitus was defined as current use of oral antidiabetic medications or insulin, fasting serum glucose > 6.5 mmol/L after fasting for a minimum of 8 h, and/or a self-reported history of diabetes (25). Smokers were considered those participants with a current history of smoking or reported a lifetime consumption of >100 cigarettes (26, 27).

### Coronary Angiography and Myocardial Revascularization

Between January 2011 and July 2020, coronary angiography was performed for all patients at admission using digital subtraction angiography. The physician used the Seldinger method to puncture the radial artery or femoral artery and applied the Judkins method to perform left and right coronary angiography. The presence of arterial stenosis was examined from four major coronary artery branches (left main, left anterior descending, left circumflex, and right coronary artery). The degree of stenosis was graded based on the presence of a plaque and stenosis of the lumen (28). The myocardial revascularization in ACS patients was obtained based on the ESC/EACTS Guidelines on myocardial revascularization (29–31). A severe coronary artery disease was defined as at least 1 coronary stenosis of more than 75%. According to the hospital protocol, two experienced interventional cardiologists who were blinded to the clinical data should review the coronary angiography results. If there was any disagreement between the two interventional cardiologists, images were reviewed and adjudicated by a senior interventional cardiologist to reach a consensus before the data recorded in the EMRRD.

### Statistical Analysis

Data were analyzed using SPSS version 23.0 (SPSS Inc. Chicago, IL, USA). The study population was stratified into quartiles based on gender-specific SUA levels and tertiles based on LDL, Lp(a), and HDL tertiles. The respective cut-off values of SUA (Q1, Q2, Q3, and Q4), LDL (T1, T2, and T3), Lp(a) (T1, T2, and T3), and HDL (T1, T2, and T3) in males and females are given in the footnote of each table. Test variables were summarized using mean ± SD for continuous variables, and percentiles for categorical variables. Student *t*-tests were performed to compare the normally distributed data. When variables were non-normal distributions, the Mann-Whitney *U*-test was applied and expressed as median (Interquartile range). The Chi-square test (χ2) was used to analyze categorical data. Binary logistic regression models were performed, and the odds ratios (OR) at 95% confidence intervals (CIs) were utilized to approximate the associated risk of UA and lipid profile for myocardial revascularization, with the lowest quartile/tertile serving as the reference category. Pearson correlation coefficient were used to evaluate the linear relationship between uric acid and lipid profiles among ACS patients. A two-sided *P* < 0.05 was considered statistically significant.

## Results

### Baseline Characteristics of the Participants

This study included 14,234 patients. Baseline demographic and clinical characteristics according to quartiles of serum UA are listed in Table 1. The mean values of SUA, Lp(a), TC, LDL were significantly higher in Q4 than in Q1–Q3. However, the mean values of creatinine and HDL levels were significantly lower in Q1 than in Q2–Q4. Patients with elevated SUA were younger (*P* < 0.001), with higher proportion of smokers (*P* = 0.017), alcohol drinkers (*P* = 0.022), but with lower prevalence of hypertension (*P* < 0.001), diabetes mellitus (*P* < 0.001). Patients in the first quartile were more often in therapy with myocardial revascularization at admission (*P* < 0.001). Patients in Q1of SUA with significant CAD at coronary angiography had a more frequent involvement of LCX (*P* < 0.001), LAD (*P* < 0.001), and RCA (*P* < 0.001). Patients in Q2 of SUA had a more frequent involvement of LCA (*P* < 0.001).

**Table 1:** Baseline characteristics of the participants.

**Variables**	**Q1 (***N*** = 3,579)**	**Q2 (***N*** = 3,561)**	**Q3 (***N*** = 3,550)**	**Q4 (N = 3,544)**	***P*** **-value**
Age (year)	64 (58-72)	63 (57-72)	62 (57-71)	62 (56-71)	<0.001
**Gender**					0.976
Male	2,556 (71.4%)	2,530 (71.0%)	2,538 (71.5%)	2,524 (71.2%)	
Female	1,023 (28.6%)	1,031 (29%)	1,012 (28.5%)	1,020 (28.8%)	
**History**					
Smoking, *n* (%)	1,410 (39.4%)	1,482 (41.6%)	1,495 (42.1%)	1,522 (42.9%)	0.017
Drinking, *n* (%)	718 (20.1%)	762 (21.4%)	798 (22.5%)	808 (22.8%)	0.022
Hypertension, *n* (%)	1,765 (49.3%)	1,558 (43.8%)	1,578 (44.5%)	1,536 (43.3%)	<0.001
Diabetes mellitus, *n* (%)	986 (27.5%)	870 (24.4%)	806 (22.7%)	758 (21.4%)	<0.001
BMI	25.97 (23.44-28.73)	26.01 (23.44-28.63)	25.91 (23.43-28.45)	26.12 (23.44-28.89)	0.092
**Biochemistry**					
LDL (mmol/L)	2.47 (1.94-2.96)	2.33 (1.73-2.89)	2.47 (1.86-2.97)	2.50 (1.97-2.97)	<0.001
HDL (mmol/L)	1.05 (0.90-1.26)	1.08 (0.90-1.29)	1.08 (0.91-1.29)	1.08 (0.91-1.28)	<0.001
TC (mmol/L)	4.29 (3.63-5.17)	4.40 (3.65-5.32)	4.37 (3.65-5.28)	4.43 (3.66-5.33)	0.001
TG (mmol/L)	1.40 (1.01-2.04)	1.45 (1.02-2.10)	1.44 (1.04-2.12)	1.41 (1.0-2.10)	0.241
LP (a) (mmol/L)	148 (83-280.5)	160 (87.15-315)	161.95 (90.175-312.1)	170 (92.05-315)	<0.001
SUA (μmol/L)	247 (220-268)	313 (299-327)	368 (354-383)	451 (423-493)	<0.001
Creatinine (μmol/L)	68 (58-79)	69 (59-78)	69 (59-80)	69 (59-80)	<0.001
Glucose (mmol/L)	5.41 (4.83-6.79)	5.35 (4.79-6.60)	5.32 (4.78-6.40)	5.29 (4.76-6.43)	<0.001
White blood cells (10^∧^9/L)	6.78 (5.53-8.51)	6.86 (5.59-8.56)	6.86 (5.59-8.64)	6.88 (5.56-8.66)	0.305
Platelets count (10^∧^9/L)	204 (168-244)	204 (171-242)	205 (172-244)	205 (170-243)	0.748
NLR	2.08 (1.14-3.58)	2.02 (1.06-3.38)	2.04 (1.12-3.31)	2.06 (1.10-3.41)	0.462
**Therapy before admission**					
Diuretics use, *n* (%)	1,605 (44.8%)	1,587 (44.6%)	1,619 (45.6%)	1,542 (43.5%)	0.356
Statin use, *n* (%)	2,982 (83.3%)	2,976 (83.6%)	3,012 (84.8%)	2,956 (83.4%)	0.262
**Therapy at admission**					
Myocardial revascularization	2,522 (70.5%)	2,172 (61%)	2,103 (59.2)	2,021 (57%)	<0.001
**Coronary artery distribution of significant CAD**					
LCA, *n* (%)	378 (10.6%)	400 (11.2%)	349 (9.8%)	353 (10%)	<0.001
LAD, *n* (%)	2,692 (75.2%)	2,486 (69.8%)	2,417 (68.1%)	2,429 (68.5%)	<0.001
LCX, *n* (%)	2,034 (56.8%)	1,885 (52.9%)	1,741 (49%)	1,753 (49.5%)	<0.001
RCA, *n* (%)	2,297 (64.2%)	2,121 (59.6%)	2,079 (58.6%)	2,022 (57.1%)	<0.001

*SUA quartiles for male = Q1, ≤ 288 μmol/L; Q2, 288–343 μmol/L, and Q3, 343-405 μmol/L; Q4, >405 μmol/L. Female = Q1, ≤ 275 μmol/L; Q2, 275–330 μmol/L, and Q3, 330-391 μmol/L; Q4, >391 μmol/L; LDL, low-density lipoprotein; HDL, high-density lipoprotein; TC, Total Cholesterol; TG, triglyceride; SUA, Serum uric acid; NLR, Neutrophil-Lymphocyte Ratio; LCA, Left coronary artery; LAD, Left anterior descending; LCX, Left circumflex artery; RCA, Right coronary artery*.

### The Prevalence and Risk Estimate of Myocardial Revascularization Based on the Quartiles of SUA and Tertiles of LDL/Lp(a)/HDL

**Figure 2:**
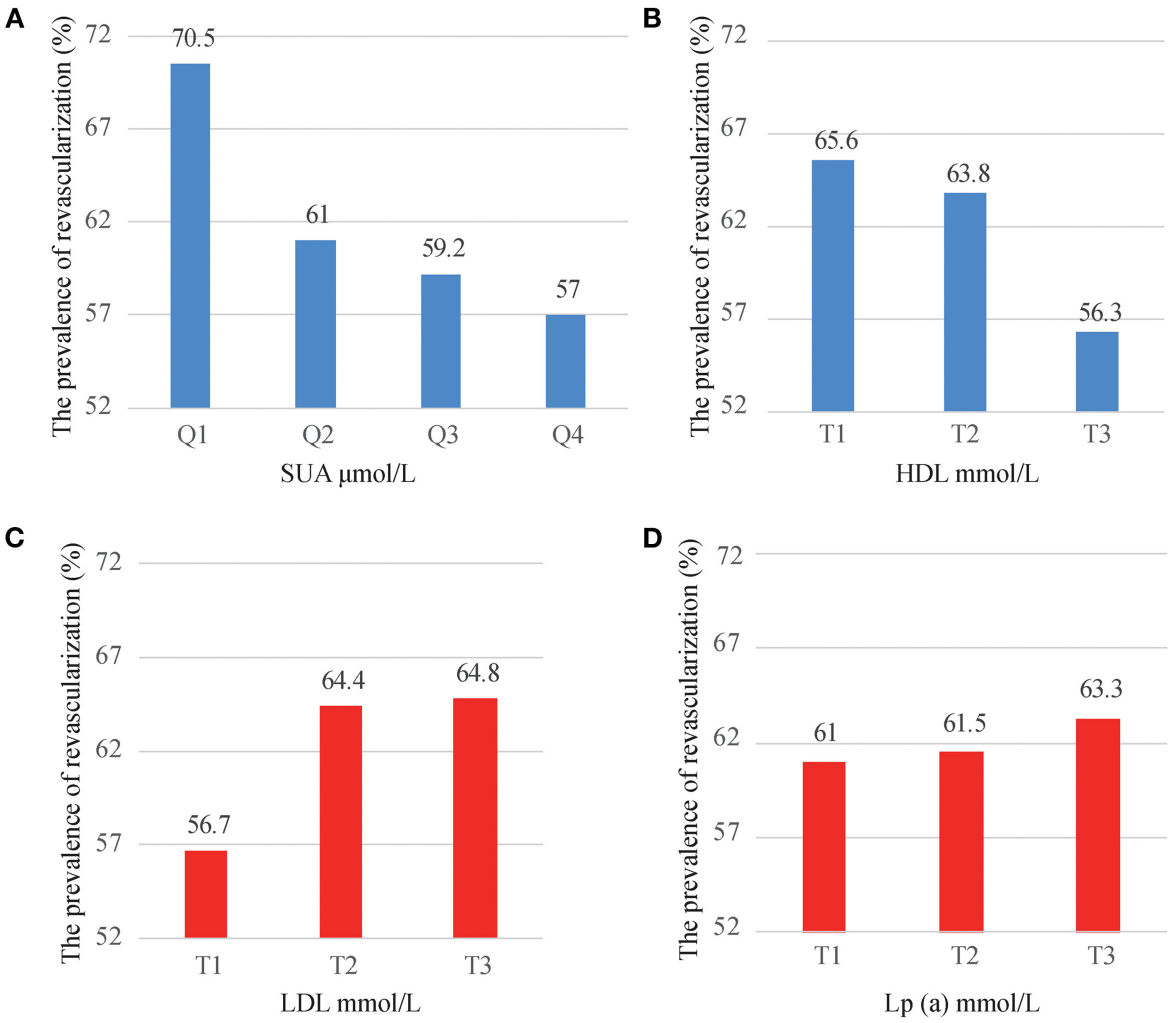
The prevalence of myocardial revascularization grouped by SUA quartiles (**A**), HDL tertiles (**B**), LDL tertiles (**C**), and Lp(a) tertiles (**D**).

Overall, myocardial revascularization was implemented in 8,818 (61.9%) patients out of 14,234 patients. The prevalence of myocardial revascularization was significantly decreased from 70.5% in the first quartile to 57% in the fourth quartile of SUA, respectively (Figure 2A). Based on HDL tertiles, the prevalence of myocardial revascularization was also significantly decreased from 65.6 to 56.3% with an increase in HDL tertiles (Figure 2B). Myocardial revascularization, on the other hand, increased considerably from 56.7% in the low tertile to 64.4 and 64.8% in the middle and upper tertiles of LDL, respectively (Figure 2C). Likely, the prevalence of myocardial revascularization increased considerably from 61% in the low tertile of Lp(a) to 61.5 and 63.3 in the middle and upper tertiles, respectively (Figure 2D).

Table 2 presents the ORs of myocardial revascularization among ACS patients grouped by the quartiles of SUA levels. We observed that, compared with the first quartile of SUA, the risk association of myocardial revascularization was decreased with the increase of SUA levels. This decrease in risk of myocardial revascularization persisted even after adjusting for age, gender, smoking, HDL, LDL, TG, Glucose, Creatinine, statin use, hypertension. Compared with the first quartile, the adjusted OR (95%CI) for the patients in Q2, Q3, and Q4 were 0.614 (0.528-0.714), 0.551 (0.474-0.639) and 0.477 (0.411-0.554), respectively (*P* for trend <0.001).

**Table 2:** The risk estimate for the myocardial revascularization based on the SUA quartiles, HDL tertiles, LDL tertiles, and Lp (a) tertiles.

	**SUA quartiles (μmol/L)**			
	**Q1**	**Q2**	**Q3**	**Q4**
Unadjusted model	Ref.	0.655 (0.594-0.723)^‡^	0.609 (0.552-0.672)^‡^	0.556 (0.504-0.613)^‡^
Adjusted model	Ref.	0.614 (0.528-0.714)^‡^	0.551 (0.474-0.639)^‡^	0.477 (0.411-0.554)^‡^
	**LDL tertiles (mmol/L)**			
	**T1**	**T2**	**T3**	
Unadjusted model	Ref.	1.385 (1.275-1.504)^‡^	1.407 (1.295-1.529)^‡^	
Adjusted model	Ref.	1.862 (1.646-2.106)^‡^	2.053 (1.812-2.326)^‡^	
	**Lp(a) Tertiles (mmol/L)**			
	**T1**	**T2**	**T3**	
Unadjusted model	Ref.	1.022 (0.941-1.110)	1.105 (1.017-1.201)*	
Adjusted model	Ref.	1.102 (0.973-1.248)	1.204 (1.062-1.365)^†^	
	**HDL tertiles (mmol/L)**			
	**T1**	**T2**	**T3**	
Unadjusted model	Ref.	0.924 (0.850-1.005)	0.675 (0.621-0.733)^‡^	
Adjusted model	Ref.	0.944 (0.860-1.036)	0.884 (0.804-0.972)*	

* *P < 0.05*,

† *P < 0.01*,

‡ *P < 0.001*.

*SUA-quartile 1, male ≤ 288 μmol/l, female ≤ 275 μmol/l; SUA-quartile 2, male 288–343 μmol/l, female 275–330 μmol/l and SUA-quartile 3, male 343-405 μmol/l, female 330-391 μmol/l; SUA-quartile 4, male > 405 μmol/l, female > 391 μmol/l; Adjusted for age, gender, smoking, HDL, LDL, TG, Glucose, Creatinine, Statin use, Hypertension. LDL-tertile 1, male ≤ 2.08 mmol/l, female ≤ 2.11 mmol/l; LDL-tertile 2, male 2.08–2.76 mmol/l, female 2.11–2.81 mmol/l and LDL-tertile 3, male > 2.76 mmol/l, female > 2.81 mmol/l; Adjusted for age, gender, smoking, HDL, UA, TG, Glucose, Creatinine, Statin use, Hypertension. Lp (a)-tertile 1, male ≤ 108.5 mmol/l, female ≤ 109.2 mmol/l; Lp (a)-tertile 2, male 108.5–240 mmol/l, female109.2–249 mmol/l; and Lp (a)-tertile 3, male > 240 mmol/l, female > 249 mmol/l; Adjusted for age, gender, smoking, HDL, LDL, TG, UA, Glucose, Creatinine, Statin use, Hypertension. HDL-tertile 1, male ≤ 0.96 mmol/l, female ≤ 0.96 mmol/l; HDL-tertile 2, male 0.96–1.21 mmol/l, female 0.96–1.18 mmol/l; and HDL-tertile 3, male > 1.21 mmol/l, female > 1.18 mmol/l; Adjusted for age, gender, smoking, LDL, TG, UA, Glucose, Creatinine, Statin use, Hypertension*.

Compared with the first tertile of LDL, the risk association of myocardial revascularization was increased with the increase of LDL levels. The risk association remained after adjustment for potential confounders including as age, gender, smoking, HDL, SUA, TG, Glucose, Creatinine, statin use, and hypertension. The adjusted OR (95% CI) of myocardial revascularization in 3rd tertile compared to the 1st tertile of LDL was 2.053 (1.812, 2.326; *P* < 0.001). When grouped by Lp(a) tertiles, the third tertile of Lp(a) levels was associated with a 1.204-fold increased risk of myocardial revascularization compared to those in the 1st tertile of Lp(a) after adjusting for a variety of confounding variables [the adjusted OR (95% CI) = 1.204 (1.062, 1.365; *P* = 0.004)] including, age, gender, smoking, HDL, LDL, TG, SUA, Glucose, Creatinine, statin use, hypertension. After adjusting for several confounding factors, including, age, gender, smoking, LDL, TG, SUA, Glucose, Creatinine, statin use, and hypertension, the third tertile of HDL was linked with a 0.884-fold lower risk of myocardial revascularization compared to those in the first tertile of HDL [the adjusted OR (95% CI) = 0.884 (0.804, 0.972; *P* = 0.011)] (Table 2). However, as shown in Supplementary Table 1, the linear relationships between uric acid and lipid profiles among ACS patients were not significant.

### The Effect of SUA and LDL Interaction in Myocardial Revascularization

**Figure 3:**
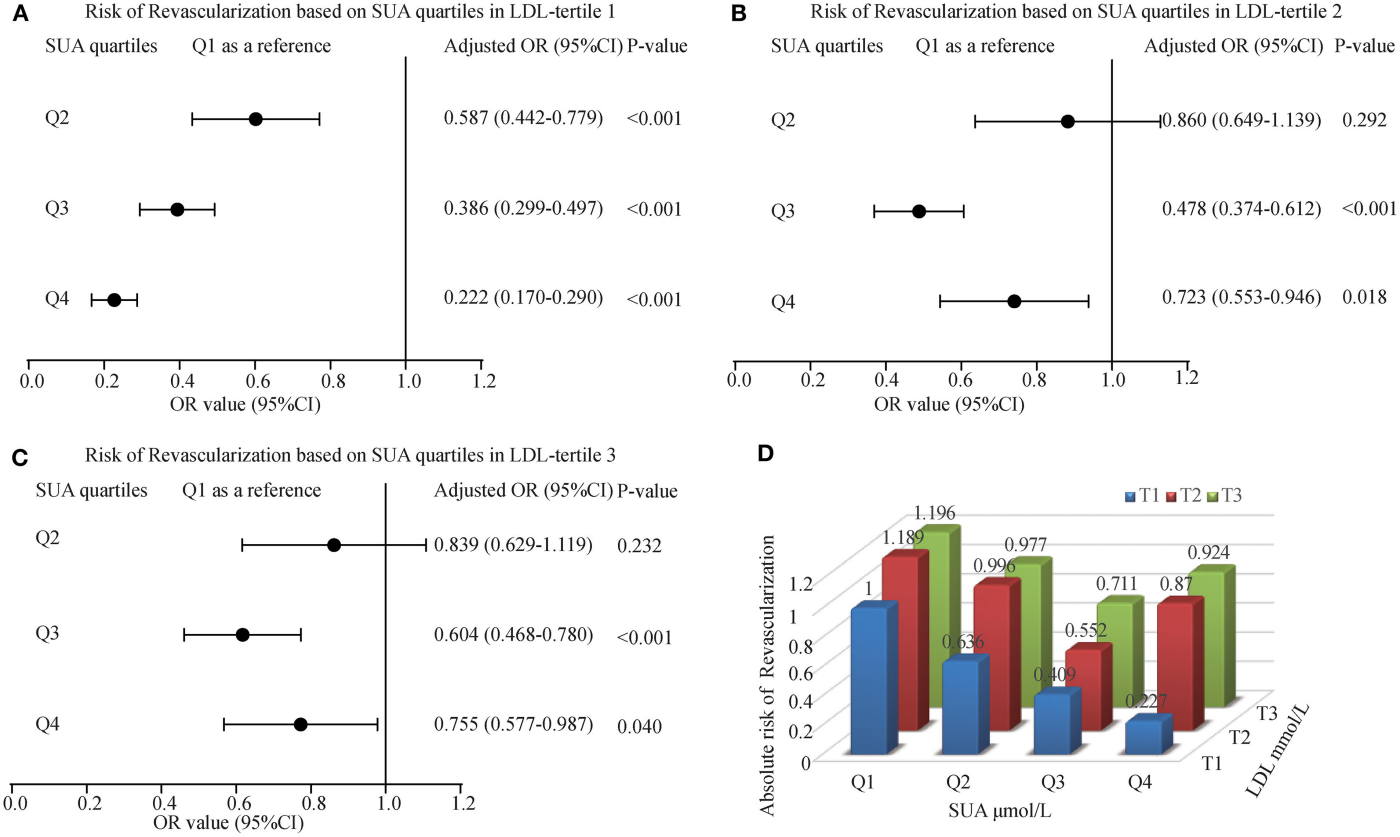
The risk of myocardial revascularization (MRT) based on baseline SUA quartiles in ACS patients. (**A**) Adjusted odd ratios for MRT in LDL-tertile 1; (**B**) Adjusted odd ratios for MRT in LDL-tertile 2; (**C**) Adjusted odd ratios for MRT in LDL-tertile 3; (**D**) Adjusted odd ratios for MRT in each group by quartiles of the SUA and tertiles of the LDL. Adjusted for age, gender, smoking, HDL, TG, Glucose, Creatinine, Statin use, and Hypertension.

To calculate the interaction impact between SUA and LDL, we estimated the OR and 95% CI for myocardial revascularization in those patients grouped by LDL tertiles along with their matched SUA quartiles. The regression analysis indicated that patients in the first tertile of LDL had a gradually lower risk of myocardial revascularization as their SUA levels increased. Also, ACS patients at the higher quartiles of SUA had a lower risk for myocardial revascularization across second and third tertiles of LDL. The lowest OR (95% CI) for the subjects in tertiles 1, 2, and 3 were 0.222 (0.170-0.290), 0.478 (0.374-0.612), and 0.604 (0.468-0.780), respectively. The ORs linked with an elevated SUA among participants classified by tertiles of LDL were shown in Figures 3A–C. Similarly, the patients at lower tertiles of LDL grouped by SUA shows the lowest risk of been treated with myocardial revascularization (Figure 3D).

### The Effect of SUA and Lp(a) Interaction in Myocardial Revascularization

**Figure 4:**
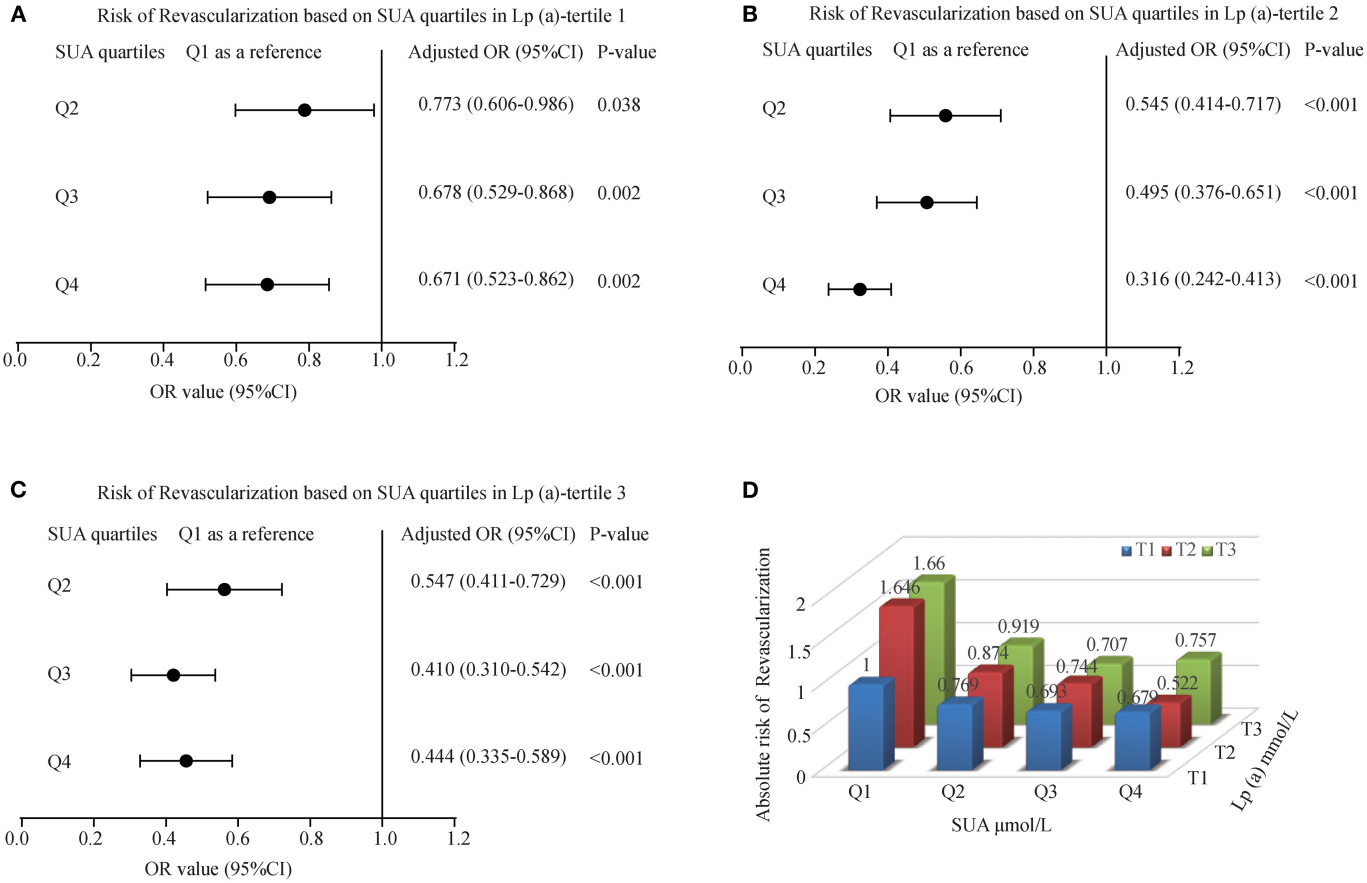
The risk of myocardial revascularization (MRT) based on baseline SUA quartiles in ACS patients. (**A**) Adjusted odd ratios for MRT in Lp(a)-tertile 1; (**B**) Adjusted odd ratios for MRT in Lp(a)-tertile 2; (**C**) Adjusted odd ratios for MRT in Lp(a)-tertile 3; (**D**) Adjusted odd ratios for MRT in each group by quartiles of the SUA and tertiles of the Lp(a). Adjusted for age, gender, smoking, HDL, LDL, TG, Glucose, Creatinine, Statin use, and Hypertension.

We explored the effect between SUA and Lp(a) interaction in myocardial revascularization. With an increase in SUA levels, the risk of myocardial revascularization was decreased gradually among patients present in T1 of Lp(a) category (OR = 0.773, 0.678, 0.671 for Q2, Q3, and Q4 of SUA) and T2 of Lp(a) category (OR = 0.545, 0.495, 0.316 for Q2, Q3, and Q4 of SUA). The forest plots presented the ORs associated with an elevated SUA in participants grouped by Lp(a) tertiles are illustrated in Figures 4A–C. However, at the T3 Lp(a) level, the third quartile of SUA has the lowest risk [OR (95%CI) = 0.410 (0.310-0.542)]. Likewise, patients in the lower tertiles of Lp(a) grouped by SUA also had a decreased likelihood of undergoing MRT, with patients in T1 having the lowest risk (OR = 0.769, 0.693, and 0.679 for Q2, Q3, and Q4, respectively) (Figure 4D).

### The Effect of SUA and HDL Interaction in Myocardial Revascularization

**Figure 5:**
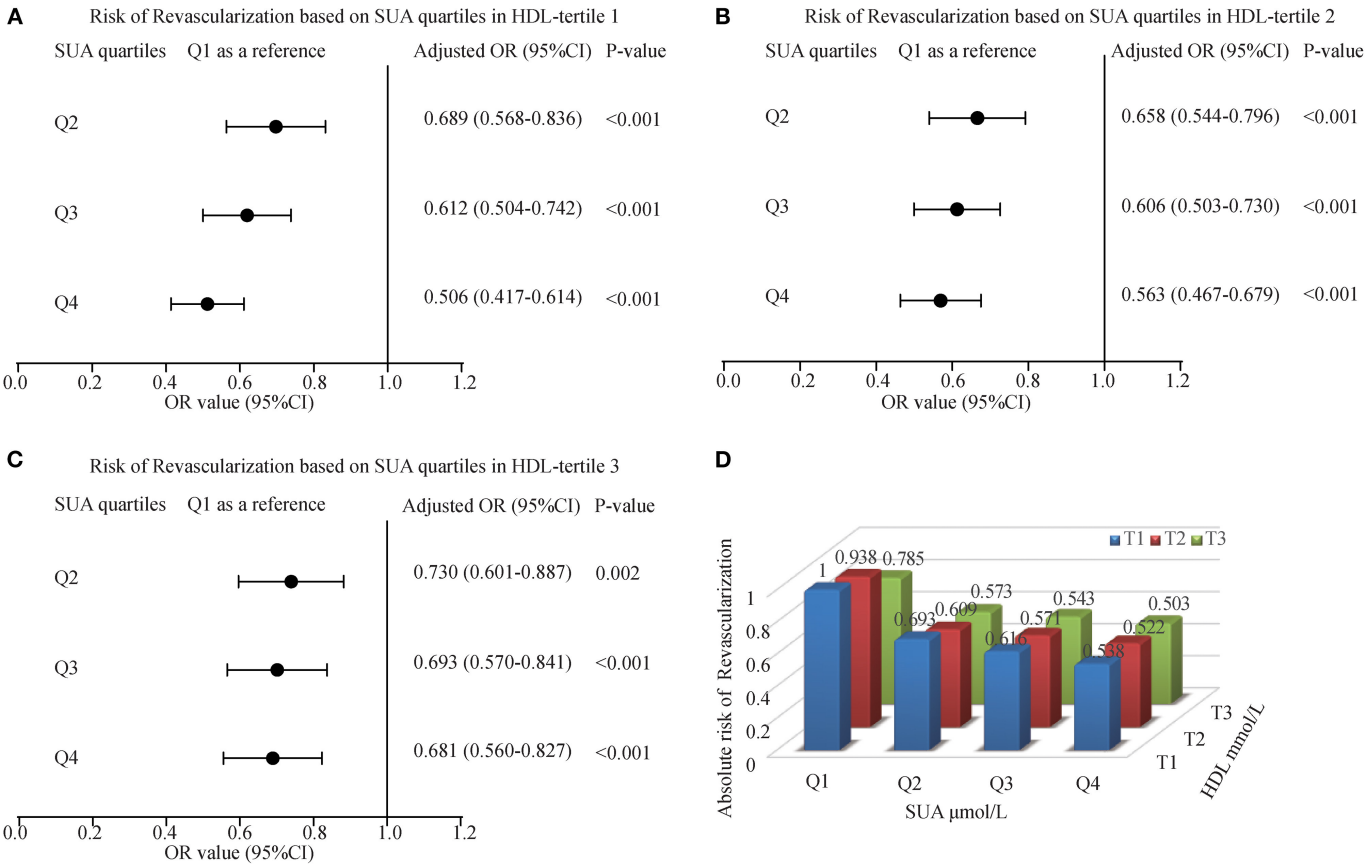
The risk of myocardial revascularization (MRT) based on baseline SUA quartiles in ACS patients. (**A**) Adjusted odd ratios for MRT in HDL-tertile 1; (**B**) Adjusted odd ratios for MRT in HDL-tertile 2; (**C**) Adjusted odd ratios for MRT in HDL-tertile 3; (**D**) Adjusted odd ratios for MRT in each group by quartiles of the SUA and tertiles of the HDL. Adjusted for age, gender, smoking, LDL, TG, Glucose, Creatinine, Statin use, and Hypertension.

Figure 5 showed the interaction effect between elevated SUA and HDL. With an increase in SUA levels, the risk of myocardial revascularization was decreased gradually when ACS patients were grouped based on HDL tertiles. The ORs (95% CI) for MRT in the tertiles of HDL for those patients in the fourth quartile of SUA were 0.506 (0.417-0.614), 0.563 (0.467-0.679), and 0.681 (0.560-0.827), respectively. Figure 5D likewise, illustrated that the highest quartile of SUA levels had the lowest risk of undergoing MRT, with patients in Q4 having the lowest risk of MRT (OR = 0.538, 0.522, and 0.503 for T1, T2, and T3, respectively).

### The Impact of Serum Uric Acid in Myocardial Revascularization in ACS Patients Without DM

**Figure 6:**
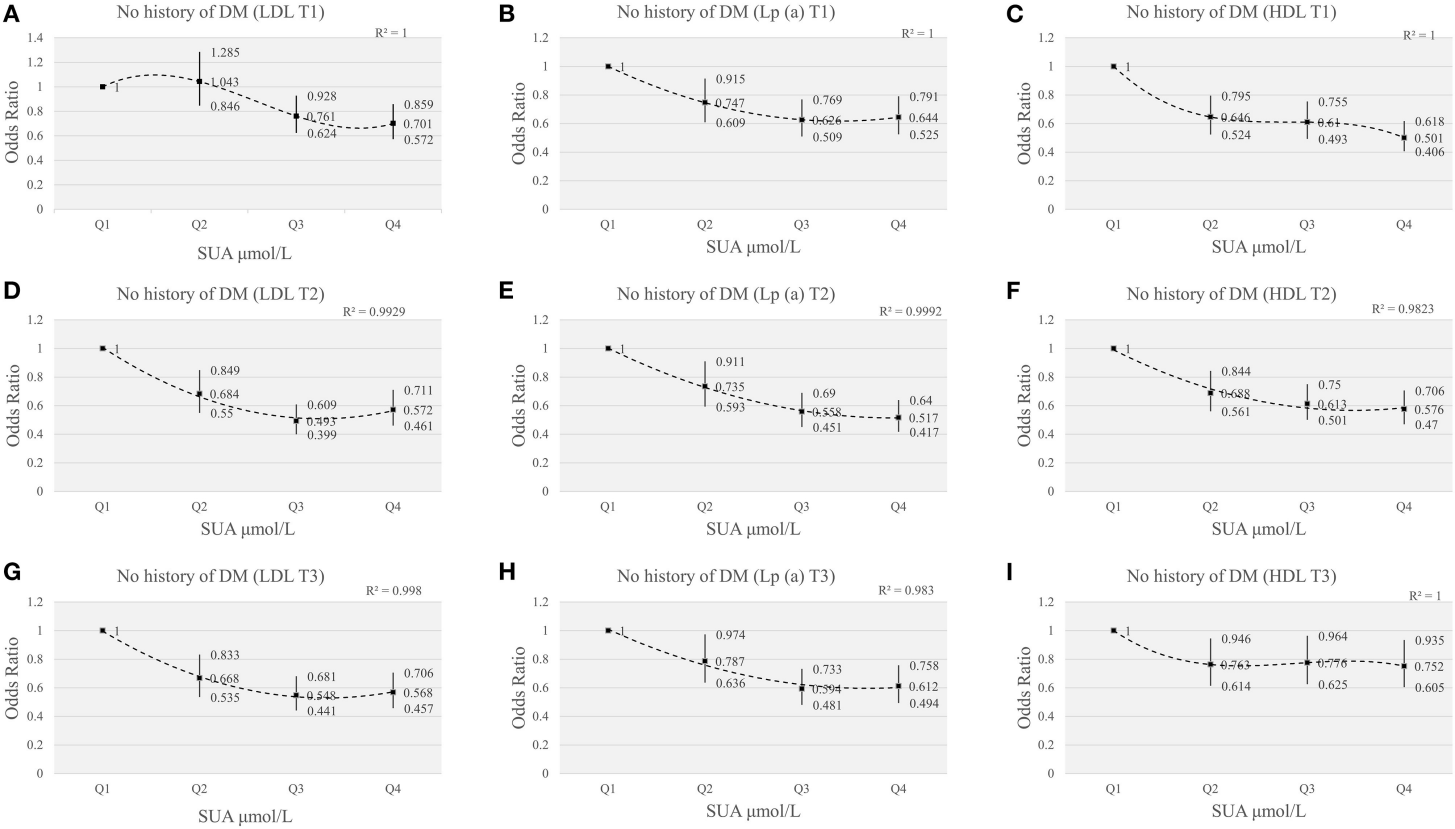
The risk of myocardial revascularization based on SUA quartiles grouped by LDL/Lp(a)/HDL tertiles in ACS patients without DM. **(A,D,G)** adjusted for age, gender, smoking, HDL, TG, Glucose, Creatinine, Statin use, Hypertension; **(B,E,H)** adjusted for age, gender, smoking, HDL, LDL, TG, Glucose, Creatinine, Statin use, Hypertension; **(C,F,I)** adjusted for age, gender, smoking, LDL, TG, Glucose, Creatinine, Statin use, Hypertension.

To better confirm the effect of SUA level on myocardial revascularization, we further analyzed ACS patients without diabetes. Compared with the first quartile of SUA levels, the risks of myocardial revascularization were significantly lower in different tertiles of lipids components (LDL, Lp(a), HDL). Figure 6 showed the interaction effect of elevated SUA and lipids, including LDL, Lp(a), HDL. Figures 6A,D,G showed that the lowest ORs (95%CI) for myocardial revascularization in SUA quartiles categorized by LDL tertiles were 0.701 (0.572, 0.859) for Q4 of T1, 0.493 (0.399, 0.609) for Q3 of T2, 0.548 (0.441-0.681) for Q3 of T3. Similarly, in Lp(a) tertiles, the lowest ORs (95%CI) for myocardial revascularization associated with SUA were 0.626 (0.509, 0.769) for Q3 of T1, 0.517 (0.417, 0.640) for Q4 of T2, and 0.594 (0.481-0.733) for Q3 of T3 (Figures 6B,E,H). When the patients were categorized based on HDL tertiles, those participants present at the highest quartile of SUA levels had the lowest risk of myocardial revascularization (Figures 6C,F,I). After excluding patients with diabetes mellitus, our results showed that the increase of SUA was still associated with the decreased risk of myocardial revascularization at different concentrations of lipid indices, suggesting that DM status did not influence the outcome of the data.

## Discussion

In this retrospective study, elevated SUA and HDL were found to be inversely linked with an increased chance of MRT during admission of 14,234 ACS patients from the hospital registry. Whereas, LDL and Lp(a) levels were positively associated with MRT among ACS patients. Also, the interaction analyses between SUA and lipid profiles, particularly [LDL, and Lp(a)] show that patients in higher SUA quartiles grouped by the lipid components had a significantly decreased chance of undergoing MRT. Even after the adjustment for possible confounders, this risk association of SUA and lipid components with MRT continues.

In this study, our data suggested that elevated SUA were associated with a decreased likelihood of MRT among ACS patients. In the past, high levels of SUA was speculated to increase mortality and major adverse cardiovascular events in patients with acute myocardial infarction (32). On the contrary, a recent study by Zhang et al. reported that SUA (300 μmol/L) can alleviate the damage of acute hypoxia (33). This indicates the theory behind SUA is paradoxical in view of its association with acute and chronic CAD. More and more new clinical studies have found that uric acid has a neuroprotective effect. Each milligram per deciliter increase in blood uric acid increases the probability of excellent clinical outcomes by 12% in patients with acute ischemic stroke (34). Brouns et al. found that lower UA during the first week after stroke was associated with a more severe stroke, poor stroke evolution, and poor long-term stroke prognosis (35). A clinical study on the Chinese population demonstrated that a proper increase of serum uric acid is conducive to the prognosis of young patients with cerebral infarction (36). Uric acid is also beneficial to the prognosis of cerebral infarction, according to recent investigations by Wu et al. (37) and Liu et al. (38). The available clinical trial data supporting UA as a form of neuroprotective drug for ischemic stroke was summarized by Li et al. (39). Therefore, it is important to explore the relationship between the increase of SUA levels in patients with acute ACS and myocardial revascularization.

The role of oxidative stress in the development of atherosclerosis cannot be overstated (40). Interestingly, UA contributes up to 60% of free-radical scavenging in human serum (41), and its potential as a medicinal antioxidant has already been noted (42). Our study found that ACS patients with high SUA values, regardless of their different categories of lipid profile, had a lower likelihood of undergoing MRT. According to the previous studies, acute oxidative stress due to tissue ischemia and cell redox state alteration occurs in ischemic stroke, acute lower limb ischemia, and myocardial infarction (43–46). The subsequent elevation of SUA concentration due to ischemic tissue metabolism of adenosine (47–49), loss of nitric oxide-induced inhibition of xanthine oxidase (50), and impaired oxidative metabolism (51) are all similar findings in these states. In this regard, SUA increase could be a preventive response to counteract the negative consequences of free radical activity and oxidative stress.

High levels of LDL cholesterol and Lp(a) have a strong correlation with CAD, especially in acute myocardial infarction (52–54). Also, Lp(a) is considered not only a risk factor for atherosclerotic diseases but also speculated to closely associate with the pathophysiology of thrombotic diseases (55). Furthermore, an epidemiological study found that Lp(a) > 30 mg/dl was linked to an increased risk of coronary heart disease (52). On the contrary, CAD is negatively associated with low HDL cholesterol. Importantly, lipid-lowering therapy has proven to significantly reduce the morbidity and mortality of CVDs, which confirm the deleterious effect of hypercholesterolemia. In the present study, our findings show that high LDL and Lp(a) levels were positively associated with MRT among ACS patients, whereas HDL-c was negatively associated with MRT. But, the risk of MRT decreased when participants were categorized based on their SUA levels, even among the subjects present at the highest tertiles of LDL-c and Lp(a).

We investigated the relationship between SUA level and lipid indices in ACS patients, taking into account the direct effect of SUA, which is known to be a product of xanthine-oxidoreductase activity, and the role of elevated LDL-c and Lp(a) in modifying the pathophysiology of ACS. Most patients in the top quartiles of SUA levels had a decreased risk of MRT, according to the interaction assessment in those patients grouped by the different lipid indices tertiles and their corresponding quartiles of SUA. These results show that a subsequent rise of SUA concentration in ACS may indicate a protective response. Because it has been demonstrated that the physiologic effect of UA can be strengthened by short-term administration to prevent oxidative and free radical-mediated tissue damage, and that early administration of mixed antioxidants improved cardiovascular hemodynamics significantly (56). Also, a shred of evidence has documented that the administration of SUA significantly increases the *ex vivo* antioxidant capacities, complying with the normal and low oxidant stress, of healthy volunteers with lower SUA baseline concentrations (4). Another study reported that long exposure to moderately high SUA concentration appears important, as the acute elevation in tumor lysis syndrome does not necessarily cause gout even at concentrations beyond 1,200 uM (57). Collectively, these pieces of evidence indicate that the acute increase in SUA as a physiologic response can play an important role in maintaining hemostatic equilibrium, presumably via the ROS pathway. Therefore, the observed lower risk of MRT among ACS patients present at the top quartiles of SUA (despite the high levels of lipid profile/indices) confirms that the acute elevation of SUA may play a protective role in ACS patients in a short time.

Hyperuricemia and dyslipidemia often coexist in CVD patients (8–10). Paradoxically, dyslipidemia is more often due to other conditions, like hypertension or diabetes than SUA itself, in the presence of chronic hyperuricemia. Chronic oxidative stress is present in patients with hypercholesterolemia, diabetes, and hypertension, and it is speculated to play a key role in the development and progression of atherosclerosis (58). Compared to the above-mentioned risk factors, many researchers reported a positive association between hyperuricemia and incident DM (59–61). Therefore, to avoid selection bias, and to better confirm the effect of SUA level on myocardial revascularization, we further analyzed ACS patients without diabetes. We still observed that ACS patients free of DM with high levels of SUA had a lower likelihood of MRT.

The current study has certain advantages and disadvantages. To the best of our knowledge, no study has looked into the interaction between SUA and lipid indices in MRT with ACS using admission data. As a result, it's unclear if the association between high SUA levels and lipid indices [LDL-c, Lp(a), and HDL-c] amplifies or squeezes the risk of MRT in ACS patients. This study, however, should be interpreted with some limitations. The single-centered retrospective design has limited the cause-and-effect relationship between SUA/lipid profiles and the risk of MRT in ACS. Also, our data cannot provide data on the long-term effects of SUA on ACS prognosis. Similarly, due to the nature of our study, we do not have detailed information on the previous history of SUA, which otherwise could help to further understand the difference of SUA levels prior to ACS and during ACS occurrences.

## Conclusion

An increase in SUA levels may decrease the need for myocardial revascularization, even in those with increased Lp(a) and LDL-c. Elevated serum uric acid may play a protective role in ACS patients in the short term. Further study is required to confirm the observed association.

## Data Availability Statement

The raw data supporting the conclusions of this article will be made available by the authors, without undue reservation.

## Ethics Statement

The studies involving human participants were reviewed and approved by the First Affiliated Hospital of Dalian Medical University institutional review board. Written informed consent was not required for this study, in accordance with the local legislation and institutional requirements.

## Author Contributions

All authors listed have made a substantial, direct and intellectual contribution to the work, and approved it for publication.

## Conflict of Interest

The authors declare that the research was conducted in the absence of any commercial or financial relationships that could be construed as a potential conflict of interest.

## Publisher's Note

All claims expressed in this article are solely those of the authors and do not necessarily represent those of their affiliated organizations, or those of the publisher, the editors and the reviewers. Any product that may be evaluated in this article, or claim that may be made by its manufacturer, is not guaranteed or endorsed by the publisher.
