# Supplementary material for: The Relationship Between Serum Uric Acid at Different Concentrations of Lipid Indices and the Risk of Myocardial Revascularization in Patients With Acute Coronary Syndrome: A Retrospective Analysis
Source: Front Cardiovasc Med. 2021 Aug 23;8:732715. doi: 10.3389/fcvm.2021.732715 (PMC8419518; doi:10.3389/fcvm.2021.732715)
Supplement: Supplementary file 1 [file Table_1.DOCX]

Table S1 Pearson correlation coefficient between SUA and lipid indices

| Lipid indices | R value | P value |
| --- | --- | --- |
| LDL | 0.001 | 0.944 |
| HDL | 0.016 | 0.05 |
| LP(a) | 0.045 | < 0.001 |
